# High-Performance-Based Perovskite-Supported Nanocomposite for the Development of Green Energy Device Applications: An Overview

**DOI:** 10.3390/nano11041006

**Published:** 2021-04-14

**Authors:** Tse-Wei Chen, Rasu Ramachandran, Shen-Ming Chen, Ganesan Anushya, Selvarajan Divya Rani, Vinitha Mariyappan, Perumal Elumalai, Nagamalai Vasimalai

**Affiliations:** 1 Department of Materials, Imperial College London, London SW7 2AZ, UK; t.chen19@imperial.ac.uk; 2 Department of Chemistry, The Madura College, Vidya Nagar, Madurai 625011, India; diviselvarajan30@gmail.com; 3 Electroanalysis and Bioelectrochemistry Lab, Department of Chemical Engineering and Biotechnology, National Taipei, University of Technology, No.1, Section 3, Chung-Hsiao East Road, Taipei 106, Taiwan; vinithavicky80@gmail.com; 4 Department of Physics, S.A.V. Sahaya Thai Arts and Science (Women) College, Sahayam Nagar, Kumarapuram Road, Vadakkankulam, Tirunelveli 627116, India; g.anushya7@gmail.com; 5 Department of Green Energy Technology, Pondicherry University, Puducherry 605014, India; elumalai.get@pondiuni.edu.in; 6 Department of Chemistry, B.S. Abdur Rahman Cresecent Institute of Science and Technology, Chennai 600048, India; vasimalai@crescent.education

**Keywords:** perovskite, synthesis strategies, nanocomposite, energy-storage devices, cyclic stability

## Abstract

Perovskite-based electrode catalysts are the most promising potential candidate that could bring about remarkable scientific advances in widespread renewable energy-storage devices, especially supercapacitors, batteries, fuel cells, solid oxide fuel cells, and solar-cell applications. This review demonstrated that perovskite composites are used as advanced electrode materials for efficient energy-storage-device development with different working principles and various available electrochemical technologies. Research efforts on increasing energy-storage efficiency, a wide range of electro-active constituents, and a longer lifetime of the various perovskite materials are discussed in this review. Furthermore, this review describes the prospects, widespread available materials, properties, synthesis strategies, uses of perovskite-supported materials, and our views on future perspectives of high-performance, next-generation sustainable-energy technology.

## 1. Introduction

The first crystal structure of CaTiO_3_ perovskite was discovered in 1839 by Gastv Rose. The ideal close-packed cubic-structure perovskite chemical formula is ABX_3_, where A, B, and X are mono valent metallic cation (A = Cs^+^), divalent metallic cation (B = Sn^2+^), and nonmetallic anions (X = halide anion), respectively [1]. Notably, perovskite structures have earned considerable interest as an electrocatalyst that can be widely applied in practical applications, due to their low-cost, high dielectric constant, electrochemical measurement, wide potential window, and stability improvement [2].

The rapid progress and unique nanostructured properties of perovskite have led to exploration and significant potential applications in different energy fields such as supercapacitors [3], batteries [4], solar cells [5], and fuel cells [6]. In recent years, the scientific world has focused in the development of perovskite-based electrode materials, which are noteworthy due to energy crises [7]. To date, the most attractive feature of the perovskite-based solar cells’ power is their power conversion efficiency (PCE) values, which were increased from 2.2% to 22.1% [8]. Nan et al. have overviewed the anion-intercalation-based supercapacitor, and successfully highlighted the specific surface area, morphology, internal resistance, electrochemical performances, charge–discharge storage mechanism, energy density, power density, and electrode cyclic stability [9]. The cubic crystal structure of a meso porous solid-state symmetric capacitor cell (STO//STO) is mostly preferred to fabricate the energy-storage devices, owing to their outstanding electrochemical behavior and good capacitance values of 212.5 F g^−1^ @ 0.63 A g^−1^ and 158.5 F g^−1^ @ 2.5 A g^−1^ [10]. The lanthanum-aluminate-based reduced graphene oxide (LaAlO_3_/RGO) composites are widely deliberated electrode materials that demonstrate superior electrochemical performance in supercapacitor applications due to their hybrid structure, which leads to fast electron transport and the hierarchical RGO that prevents the aggregation of LaAlO_3_ [11]. The meso porous morphology of LaFeO_3_ perovskite has been synthesized by the sol-gel method, and exhibited a reasonable specific capacitance value of 241.3 F g^−1^ @ 1 A g^−1^ [12].

Fuel cells are the most promising green energy-storage technology due to their distinct features such as cost-effectiveness, lower emissions and high efficiency, which fulfills future global energy requirements [13]. Bin Zhu et al. have used a fuel cell that was built by placing hydrogen at the anode and oxygen at the cathode, and the electrochemical reaction that occurred resulted in electricity and water [14]. In general, the cell reactions can be represented as follows:

At anode: H_2_ -> 2H^+^ + 2e^−^ (1)

At cathode: 1/2O_2_ + 2e^−^ -> O^2−^ (2)

Net reaction: H_2_ + 1/2O_2_ -> 2H^+^ + O^2−^ 2H^+^ + O^2−^ -> H_2_O(I) (3)

In general, fuel cells face some drawbacks like high cost, environmental pollution, lower anodic catalytic activity, and poor cyclic stability [15]. Very recently, Song et al. developed protonic ceramic fuel cells (PCFCs) that were prepared by a self-assembling process. In this context, a rationally designed BaCo_0.7_(Ce_0.8_Y_0.2_)_0.3_O_3−δ_ composite could greatly increase the number of active sites, and significantly influenced the fuel cell’s operational stability [16]. A more active site of NdFeO_3_ perovskite catalyst has also been reported, and exhibited favorable activity for direct methanol alkaline fuel cell (DMFC) applications [17]. Perovskite is currently one of the key electrode materials that are highly useful for the designing and development of an efficient solar-energy conversion system [18]. Despite that, the aim of the research could focus on a low cost, highly conductive solar-cell device with a large surface area for the enhancement of stabilized power conversion efficiency (PCE) [19]. The thermally stabled pinhole-free ZnO-NH_4_Cl materials can exhibit high electron mobility, where the corresponding perovskite solar cells (PSCs) displayed power conversion efficiency (PCE) of 10.16% [20]. Wang and coworkers have reported room-temperature-processed, fullerene-supported TiO_2_ (R2.25-Fu/Lt-TiO_2_) electron transport layers that are prepared by a simple spin-coating method and exhibited a PCE value of 18.06% [21].

**Scheme 1:**
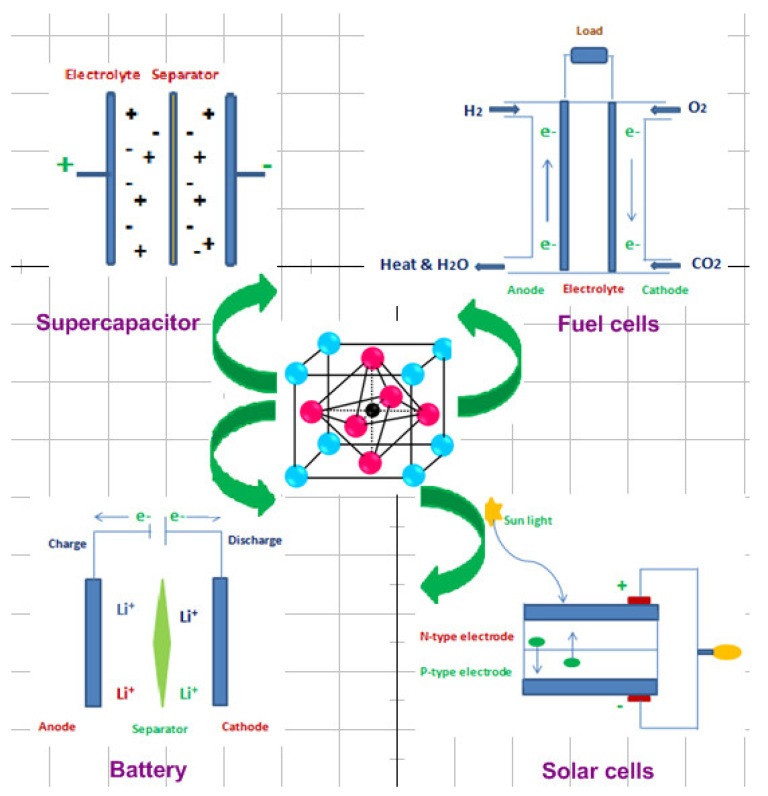
Schematic illustration of perovskite-based nanocomposite materials for energy-storage-device applications.

As a high-energy-conversion technology, solid oxide fuel cells have gained more interest owing to their solid structure, fuel flexibility, and lack of requirements for noble metals. Normally they operate from 600 to 1000 °C [22]. As a crucial material in the solid oxide fuel cell, the anode should fulfill a range of specifications, such as high electrical and ion conductivity, high (electro) catalytic activity for fuel oxidation reactions, and strong chemical stability [23]. Many researchers have proposed that anodes of a perovskite structure are good candidates for future fuel-cell anodes [24,25]. Development of an efficient, environmental-pollution-free, and inexpensive electrical energy storage (EES) device has facilitated their high energy density, high specific capacity and long cycle life [26]. Recently, Lu et al. reviewed Li-O_2_ as a promising candidate for sustainable rechargeable-battery technology. The La_2_O_2_CO_3_-La_0.7_Sr_0.3_MnO_3_ hybrid is a highly promising electrocatalyst, which can be attributed to a strong interaction between La_2_O_2_CO_3_ and La_0.7_Sr_0.3_MnO_3_ [27]. Moreover, the delivered outperforming power density value was 223.8 mW cm^−2^ [28]. Sr_0.95_Ce_0.05_CoO_3−δ_-loaded, copper-nanoparticle-based perovskite offers an alternative bi-functional electrode catalyst for rechargeable metal–air-type energy-storage devices [29]. Scheme 1 depicts the schematic illustrations of perovskite-based nanocomposite materials for various energy-device applications, such as (a) a symmetric supercapacitor, (b) a hydrogen–oxygen fuel-cell system, (c) a standard lithium-ion battery, and (d) a common solar cell.

An energy system is an apparatus used for releasing electrical energy as required. As a step to combat global warming, the role of energy-storage equipment technologies in fields such as solar energy generation and hybrid automotive systems will become increasingly important. The key focus of this review article is on supercapacitors, fuel cells, solar cells, batteries, and solid oxide fuel cells, owing to their outstanding energy platforms with considerable potential. The article further explains in detail the properties and applications of each devices.

## 2. Electrode Surface Studies

The atomic surface/surface composition study of perovskite-based electrode materials have been optimized through the low-energy ion scattering (LEIS) method. The oxide-based ABO_3_-type perovskite consists of a trivalent cation on the A site and a mixed metal valent cation on the B site. It was clearly noticed that the catalytic activity of trivalent cations was more dominated by the outer surface than the mixed valent metal cation below the surface [30]. On the other hand, Li et al. [31] overviewed the kinetics, thermodynamic studies, structural variations, and various cation surface enrichment of nanoscale-based perovskite electrode materials that achieved rational designing and influenced their electrocatalytic activity with excellent cyclic durability for more effective SOFCs. Typically, perovskite materials are known to be a prominent electrode catalyst, since they include a gas reaction, electron transfer, and active electrocatalytic activity, and have high ionic and electrocatalytic activity for SOFCs [32]. More recently, the development of a composite based on Co_3_O_4_/NiO perovskite received a great deal of attention. Modified electrode surfaces created oxygen vacancy and effectively enhanced their anion intercalation properties with high-performance energy-storage stability [33]. More importantly, the flexible and highly efficient carbon-based (graphene–Ag nanowires) PSCs have been prepared by a low-temperature method. The high quality of a decreased grain size perovskite film electrode could be attributed to light absorption and increasing the pinhole process for the optimization of commercial flexible PSCs [34]. Besides, zinc oxide-supported, Ag-NW-based flexible PSCs exhibited both optical and electrical properties, whereas the surface study of a flexible composite could decrease the surface roughness and significantly improved their remarkable flexible devices [35].

## 3. Perovskite-Based Nanocomposite in Supercapacitor Applications

There is an increasing urgent demand for the development of environmentally friendly, green, clean, and sustainable energy sources, which are widely used as the most promising energy-storage technologies [36,37,38]. Perovskite (SrCoO_3−δ_(SC), Ba_0.5_Sr_0.5_Co_0.8_Fe_0.2_O_3−δ_ (BSCF) and (Co_3_O_4_)-based electrode materials have exhibited a considerable anion-intercalation type of electrode catalyst innext-generation supercapacitor applications [39]. As for clean energy, three-dimensional perovskite NiMnO_3_ have been considered as leading the pack of emerging supercapacitor technologies, because of their cost-effectiveness and electrode stability [40]. A systematic study of anion-based pseudo-capacitance behavior has evaluated both cyclic voltammetry and charge/discharge techniques. A greater oxygen content vacancy (δ) increases the surface normalized capacitance value linearly, thereby reporting a specific capacitance value of 492 F g^−1^ [41]. In general, the electrochemical pseudo-capacitive behavior (charge/discharge method), energy density (*E*), and power density (*P*) are calculated usingthe following equations [42,43]:

*Cs* = *I* × Δ*t*/Δ*V* × *m* (4)

*E* = 1/2*C*Δ*V*^2^ (5)

**Figure 1:**
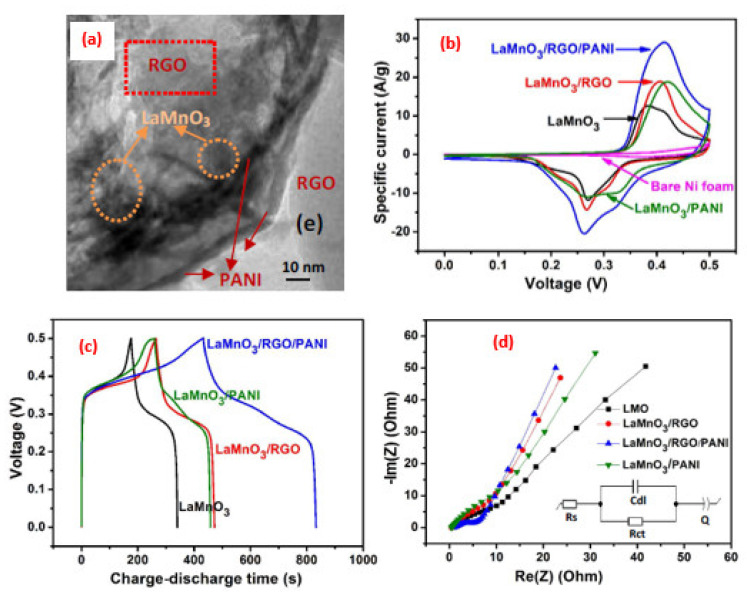
(**a**) HRTEM images of LaMnO_3_/reduced graphene oxide/polyaniline; (**b**) CV curve recorded at a scan rate of 10 mVs^−1^ for all samples and at 20 mVs^−1^ forNi foam; (**c**) Charge–discharge curve recorded for all samples at a current density of 1mA g^−1^; and (**d**) Nyquist plots for all electrodes. Copyright 2018 by the American Chemical Society [45].

*P* = *E*/Δ*t* (6)

where *C* is specific capacitance (F g^−1^), Δ*V* is potential drop (*V*), and Δ*t* is discharge time(s). An interesting supercapacitor has been fabricated using a CdS quantum-dot-based organometallic halide perovskite active electrode, and it exhibited areal capacitance of 141 μF cm^−2^, energy density of 23.8 nWh cm^−2^, and power density of 12.7 mW cm^−2^, with excellent cyclic stability (4000 cycles) [44]. Recently, well-interconnected porous LaMnO_3_/reduced graphene oxide/polyaniline ternary composite smart electrode materials have been receiving considerable attention. Figure 1a shows the surface morphology of the LaMnO_2_, PANI, and RGO composite, which was confirmed by HRTEM. The typical electrochemical voltammograms were recorded with different electrode materials at a scan rate of 10 mV s^−1^ (Figure 1b).The electrochemical charge storage-device behavior (specific capacitance value = 802 F g^−1^) shows the remarkable cyclic performances (10,000 cycles) [45] (Figure 1c,d).

**Figure 2:**
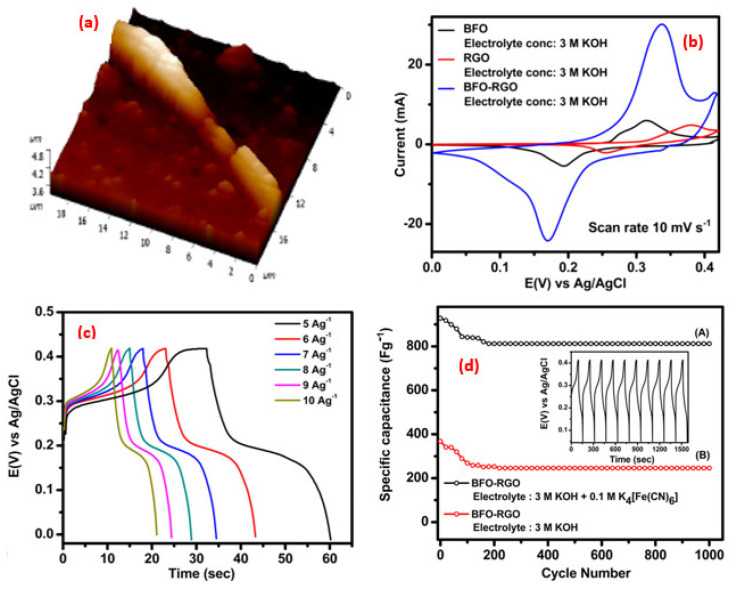
(**a**) Atomic force microscope image of BiFeO_3_ nanowire—reduced graphene oxide nanocomposite; (**b**) cyclic voltammetry curves of BiFeO_3_ nanowire, RGO, and BFO–RGO electrodes at a scan rate of 10 mV s^−1^ in 3 M KOH electrolyte; (**c**) galvanostatic charge−discharge profile of BFO–RGO electrode in 3 M KOH + 0.1 M K_4_[Fe(CN)_6_] with a changing current density of 5 to 10 A g^−1^; and (**d**) cyclic stability of the BiFeO_3_ nanowire-reduced graphene oxide electrode in (**A**) 3 MKOH + 0.1 M K_4_[Fe(CN)_6_] and (**B**) 3 M KOH showing the capacitance retention after 1000 cycles using a charge/discharge at a constant current density of 5 A g^−1^. Copyright 2019 by the American Chemical Society [63].

The two different morphological (nanofiber and nanoflake) structures of CeMO_3_ (M = Co, Ni, Cu) perovskite-type electrodes have been synthesized via the electrospinning method. The specific capacitance properties were optimized through a galvanostatic charge–discharge process under alkaline conditions, and the estimated specific capacitance values observed were 128, 189, and 117 F g^−1^, respectively [46]. A novel nanorod-like morphology of a Ba*_x_*Mn_1−*x*_O_3_ perovskite oxide electrode has attracted great attention in boosting the electrochemical properties and the favorable electrode materials for the development of supercapacitor applications (specific capacitance value of 433 F g^−1^ with good cyclic stability) [47]. Sun’s group constructed a PANI/WO_3_ composite as an active electrode material for an electrochromic supercapacitor; their common energy-storage devices can be clearly estimated by the charge–discharge process [48]. Moreover, Fan et al. [49] demonstrated an asymmetric supercapacitor withKNiF_3_ @ CNTs−8 as a positive electrode and activated carbon (AC) as a negative electrode via the solvothermal method. They achieved a better capacitive (821.4 C g^−1^ @ 1 Ag^−1^) nature with high, ultralong cyclic stability (5000 cycles). In 2017, Lang et al. studied Sr-doped lanthanum magnetite perovskite (La_1−*x*_Sr*_x_*MnO_3_, LSM) materials that delivered ahigh specific capacitance (102 F g^−1^ @ 1 A g^−1^), which was due to the redox behavior of the anion-intercalation process (Mn^2+^/Mn^3+^ and Mn^3+^/Mn) with long cycling life (5000 cycles) [50]. A meso porous, hexagonal-shaped cobalt titanate (CTO) has been explored as an efficient electrode material for supercapacitor applications, due to its tunable morphology, better capacitive behavior, and remarkable cyclic stability [51]. On the other hand, the non stoichiometric LaMn_1−*x*_O_3_ (*x* = 0, 0.05 and 0.1) perovskite materials have been synthesized by the sol-gel method. The synthesized materials demonstrated oxygen and cation vacancies, a high Mn^4+^/Mn^3+^ ratio, and a high specific capacitance value (202.1 m Ah g^−1^ 727.6 Cg^−1^) @ 1 Ag^−1^ [52]. Similarly, a simple sol-gel-assisted electrospinning technique has been used for the synthesis of a highly crystalline natured, porous SrMnO_3_ perovskite nanofiber electrode, which is associated with a reversible faradaic reaction of the active nanofiber electrode. The perovskite nanofiber exhibited electrochemical capacitance value of 321.7 F g^−1^ with better cyclic stability (5000 cycles) [53]. 3D-coral-like Ni_2_P-ACC nanostructures have been extensively used as a binder-free electrode, owing to their unique structure and larger redox active sites. The resulted areal specific capacitance value was 3352 F g^−1^ with good electrode cyclic stability (10,000 cycles) [54]. Cerium-based (CeMO_3_, M = Co, Ni, and Cu) perovskites, which have been recognized as one of the most promising candidates for the study of supercapacitor applications, demonstrated specific capacitance values of 128, 189, and 117 F g^−1^, respectively [55]. Kim et al. reported that the specific capacitance of graphene-based La_0.8_Sr_0.2_Mn_0.5_O_3−δ_ (LSMCO) G70L25 perovskite was55 F g^−1^ at a sweep rate of 5 mV s^−1^, and the electrode’s capacitance behavior curve showed a linear decrease after 4990 cycles [56]. The well flexible and paper-based microstructure natured bismuth ferrite/graphene based nanocomposite is one of the fascinating electrode materials in supercapacitor applications, the reported specific capacitance value of 9 mF cm^-2^ [57]. Silver nanoparticle-doped La_0.85_Sr_0.15_MnO_3_ has been widely studied as a promising electrode material for supercapacitors because of its high specific capacitance (186 F g^−1^) and excellent cyclic stability [58]. Ca_2.9_Nd_0.1_Co_4_O_9+δ_ perovskite has demonstrated good performance in electric double-layer capacitors, which provided a capacitance value of 53.6 F g^−1^ and an energy-density value of 16.7 Wh kg^−1^, with high electrode cyclic stability of charge–discharge [59]. Furthermore, La_0.8_Nd_0.2_Fe_0.8_Mn_0.2_O_3_-based, nitrogen-doped graphene oxide (LNFM/NGO) composites have been synthesized by the hydrothermal method, and typically the composite reveals good electronic conductivity, maximum specific capacitance (1060 F g^−1^ @ 50 mV s^−1^), and outstanding capacitive retention stability (specific capacity retention of 92.4% after 10,000 cycles) [60]. Interestingly, the active nickel-stannate-supported graphene nanosheet (NiSnO_3_/GNS) composite acts with a conducting nature for fast ion transportation during the electrochemical process, which significantly improved their specific capacitance behavior (891 F g^−1^ @ 5 mV s^−1^), with excellent cyclic charge–discharge stability (88.3% capacitance retention after 4000 cycles) [61]. The flexible electrodes of bismuthiron oxide graphene (BFO/graphene) composites have also been studied for flexible supercapacitor applications. The composite has a maximum areal capacitance of 17 mF/cm^2^ at a scan rate of 5 mV/s and degrades to 4.75 mF/cm^2^ at a scan rate of 100 mV/s [62]. Moitra et al. used the hydrothermal approach by preparing a BiFeO_3_-based reduced graphene oxide (BFO–RGO) composite for supercapacitor application. The surface roughness (nanowire) of the BFO–RGO composite was observed using an atomic force microscope (AFM) (40–200 nm) (Figure 2a). Figure 2b shows the three different cyclic voltammograms obtained for the BFO, RGO, and BFO–RGO electrodes, respectively. Figure 2c shows the CD properties of BFO–RGO, which indicated a favorable redox reaction and delivered excellent specific capacitance (526.68 F g^−1^) with good cyclic stability (1000 cycles) [63] (Figure 2).

**Figure 3:**
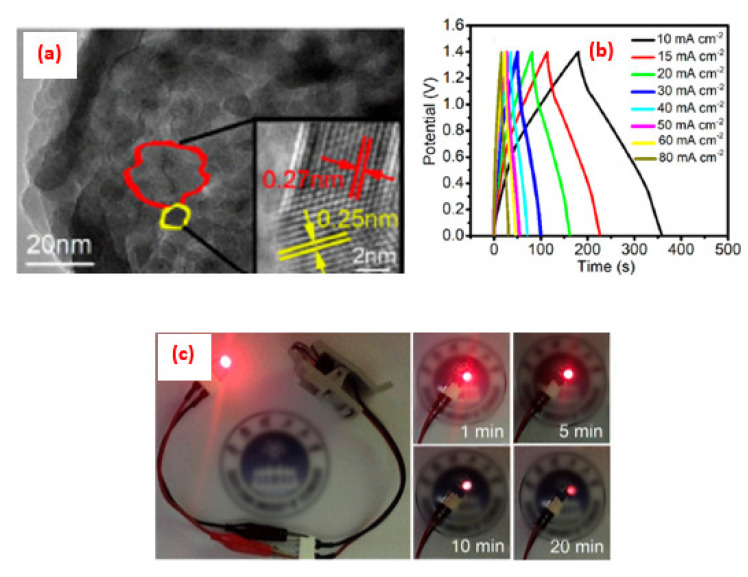
(**a**) TEM image of CuO/LSC73; (**b**) GCD curves; and (**c**) digital images of an LED powered by the two devices in series. Copyright 2019 by the American Chemical Society [64].

Liu et al. prepared a novel and binder-free CuO-modified La_1−*x*_Sr*_x_*CoO_3−δ_ cathode material to be used as a promising substrate for asymmetric supercapacitors. The detailed surface morphology and the structural entity of CuO/La_1−*x*_Sr*_x_*CoO_3−δ_ were further investigated by HRTEM (Figure 3a). Figure 3b exhibits the CD properties of a CuO/La_1−*x*_Sr*_x_*CoO_3−δ_ composite; the ceramic materials exhibited outstanding specific capacitance (545 F g^−1^) with long-term electrode stability (3500 cycles). The commercial capacitors have been tested through quasi-solid-state devices, which can shine the LED for about 20 min (Figure 3c) [64]. The energy device application of supercapacitors with different perovskite-based nanocomposite electrode materials are discussed and summarized in Table 1.

## 4. Application of Perovskite-Based Nanocomposites in Fuel Cells

**Figure 4:**
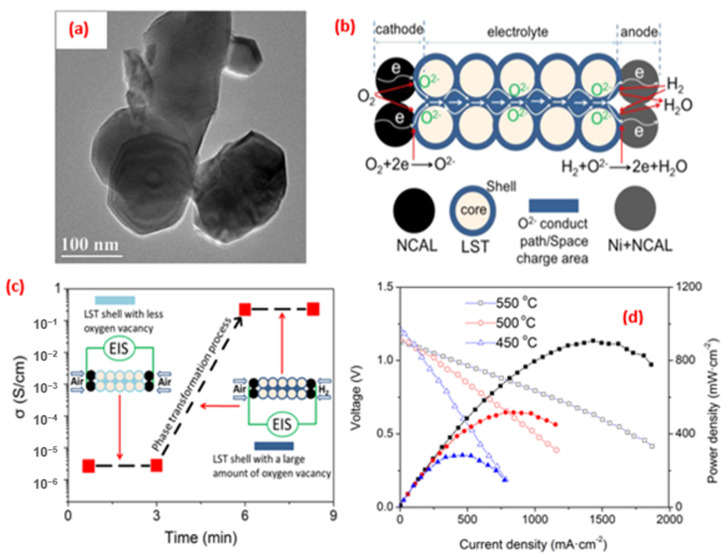
(**a**) TEM image of 2h La_0.25_Sr_0.75_TiO_3_ powder; (**b**) the electrochemical reaction mechanism; (**c**) total electrical conductivity of the cell with the LST electrolyte in air/air and in air/H_2_; and (**d**) current density-voltage/current density-power density curves of the cell operated at 550, 500, and 450 °C in H_2_. Copyright 2018 by the American Chemical Society [75].

Fuel cells have made great progress in recent decades. In reviewing the developing process of modern fuel-cell technology, it exhibited each and every important progression in technique that related to the invention of a key material. Proton exchange membrane fuel cells have been extensively studied due to their compactness and simple fabrication process, as well as their high performance, and insist to be developed commercially as automobile and stationary FC power systems. However, for more global commercialization, are duction in cost is strongly desired from the industry side. Hooshyari et al. proposed a rule-based strategy for power management to develop fuel efficiency by revolving the fuel cells in a well-organized and relevant time using predictive energy in a driving process based on La_0.9_Sr_0.1_CrO_3__−δ_-dopedperovskite nanoparticles, phosphoric acid, and poly benzimidazole for proton transfer, which increased the proton conductivity of the nanocomposite membranes and exploited the power density of 0.62 W cm^−2^ at 180 °C and 0.5 V [65]. Akhtar et al. [66] justified the graded catalyst, single-layer cubic perovskite structure La_1.0_Ba_0.5_Sr_0.5_Co_1.5_Fe_0.5_O_3−δ_ (LBSCF), which provided an optimized and non-abrupt transition in fuel-cell performance, and facilitated proton transport to regions with a high rate of oxygen reduction reaction. Furthermore, it was shown that the effects of catalyst layer grading were more pronounced at higher current densities. This was linked to the change in the spatial distribution of the reaction rate with current density, and motivates further research on catalyst layers tailored to high current density. A twin-perovskite nanocomposite cathode(TPNCC) comprising PC and MIEC nano phases with Ba–Ce–Fe–Co–O of one-pot synthesis was proposed by Zhao et al., the effects of which on the texture properties of the Ba–Ce–Fe–Co–O (BCFC) TPNCC open a new opportunity toward high-performance protonic ceramic fuel cells (PCFCs) [67]. Tang et al. developed a site-deficient layered perovskite (PrBa_0.8_Ca_0.2_)_0.95_Co_2_O_6−δ_, which indicates that the O_2_ vacancies preferentially aligned as pairs in the directions as the degree of nonstoichiometry increased; this played a central role in promoting the macroscopically observed ionic conductivity of (PrBa_0.8_Ca_0.2_)_0.95_Co_2_O_6−δ_(PBCC95) [68]. Mahimai et al. distinguished the novelty of barium strontium titanium oxide (BSTO),an attractive perovskite inorganic filler/ sulfonated poly (ether ketone) (SPEEK), polymer membrane efficiency, and adsorption strength of the membrane due to its crystalline structure, which led to high conductivity and stability [69]. Facile composite membranes consisting of SPEEK/poly (amide imide) (PAI) and SrTiO_3_-based nanocomposite electrolyte were prepared by a solvent-casting technique to exploit the combined network of SPEEK, PAI, and SrTiO_3_, owing to polymer matric of stability criteria on hydrogen bonding and excellent proton conductor, as well as being hydrophilic in nature. This offers enhanced stability and the ability to fine-tune proton conductivity in the existence of PAI and SrTiO_3_ [68,70]. Yavari et al. synthesized a PtNP–CNTs–NdFeO3NPs–CH nanocomposite in a direct methanol fuel cell (DMFC) with perovskite oxide, which exposed the multifunctional energy harvesting using a single engineered material [71]. He et al. reported the development of proton-conducting fuel cells (PCFCs) with a perovskite-type proton-conducting electrolyte P with B-site cation-deficient perovskites (BCDPs) for anode composite of the cell-component-emphasized cations, which accommodated between the inorganic layers, yielding a richer platform for chemical tuning [72]. Shao et al. demonstrated a single-layer fuel-cell design that offers an attractive possibility to diminish the reduction of power density by limiting oxygen mass transfer to the cathode and increasing the cathode-to-anode area proportion with implementation of a perovskite oxide [73]. Asghar et al. reported the solid-route and freeze-dried fuel cells, the losses of which were dramatically reduced by improving the ionic conductivity of the nanocomposite electrolytes to a record high value; i.e., ~0.55 S/cm. This breakthrough in the performance of nanocomposite fuel cells could potentially lead to commercialization of this technology [74]. Very recently, a novel core-shell, heterostructured La_0.25_Sr_0.75_TiO_3_ (LST) perovskite was introduced as an electrolyte, and is creating superionic conduction (10^−6^ S cm^−1^). The low-magnification STEM image display of LST can identify the average particle size as 100−400 nm (Figure 4a). The focused electrical conductivity was calculated using an ionic conducting mechanism by EIS studies, and they successful delivered a cell-device power-density value of 908.2 mW cm^−2^ @ 550 °C [75] (Figure 4b–d).

**Figure 5:**
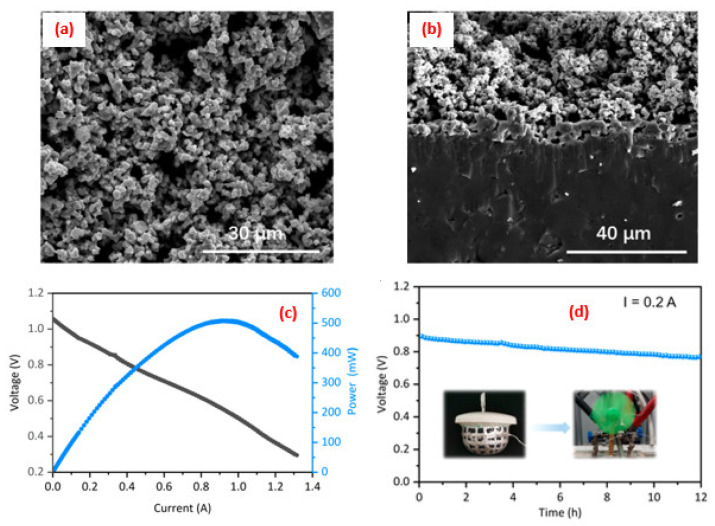
Microstructure of the (**a**) anode; (**b**) the cross-sectional image of the single cell; (**c**) current-voltage-power curve of the bowl-shaped hybrid direct carbon fuel cells and; (**d**) time-dependent voltage measured at a current of 0.2 A at 800 °C. Copyright 2020 by the American Chemical Society [76].

A new promising bowl-shaped (PrBa)_0.95_Fe_1.8−*x*_Cu*_x_*Nb_0.2_O_5+δ_ (PBFCN) perovskite cell has been prepared by a screen-printing method. Two different magnifications of the as-prepared microstructure-based porous PBFCN were observed using SEM analysis (Figure 5a,b), and the developed anode materials displayed outstanding catalytic activity under a fuel-cell condition, and the enhanced cell performance value observed was 431 mW cm^−2^ [76] (Figure 5c,d).

**Figure 6:**
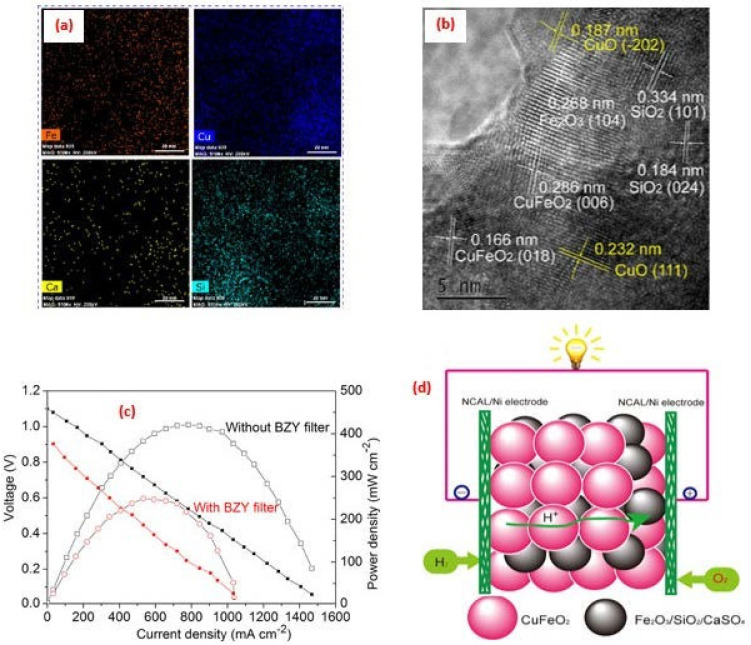
(**a**) The EDS elemental mapping (Fe, Cu, Ca, and Si elements); (**b**) HRTEM image of CuFe-oxide; (**c**) *I*–*V* and power density of CuFe-oxide cells with and without BaZr_0.8_Y_0.2_O_3-δ_ filters under H_2_/air at 550 °C; and (**d**) the underlying mechanism of the CuFe-oxide fuel cell depicted schematically. Copyright 2018 by the American Chemical Society [77].

The mineral-based, microstructure-natured CuFeO_2_-Li_x_ZnO-Sm_0.2_Ce_0.8_O_2-δ_ composite materials have been widely investigated. Figure 6a shows the ion-transfer mechanism of a CuFe-oxide fuel cell device that could promote the oxygen-reduction process and resulted in a high power output. This may enhance the ionic conductivity, and helps in the scientific understanding of the fuel-cell-performance concept from both theoretical and experimental aspects (Figure 6b–d) [77].

The preparation and analytical characterization of a PVA-supported polyvinyl pyrrolidone/BaZrO_3_ nanocomposite for constructing proton-exchange membrane fuel cells (PEMFCs) was reported. The as-prepared PVA/PVP/BaZrO_3_ nanocomposite showed proton conductivity (6.01 × 10^−2^ S cm^−1^) and the highest peak power density (28.99 mW cm^−2^) [78]. A novel nano CaTiO_3_-based poly[2,2-(m-phenylene)-5,5-bibenzimidazole] (CaTiO_3_/m-PBI) composite was prepared by the sol-gel method. The observed conductivity value was in the order of 32.7 mS cm^−1^, whereas they demonstrated a power-density value of 419 mA cm^−2^ @ 160 °C [79]. Table 2 summarizes the different types of perovskite-supported nanocomposites and their lattice parameters for fuel-cell applications.

## 5. Advance in the Power Density Level for SOFCs

The fuel cell is an electrochemical-reaction-based system capable of transforming chemical energy into high-efficiency, low-emission electrical energy [80]. Of all fuel cells, the SOFC is the most advantageous because of many benefits such as active kinetics, stable behavior, and solid structure, which can avoid all kinds of leakage and corrosion problems [81]. It is known to allow high-efficiency generation of energy from a wide range of fuels in an environmentally friendly way [82]. A standard SOFC is made up of an electrolyte, an anode, and a cathode [83]. The development of robust cathode materials for SOFCs, which operate at intermediate temperatures (500–700 °C), has attracted much attention because of their potential to dramatically reduce the cost of SOFC technology [84]. SOFCs are an excellent alternative for distributed generation in cities, as well as large-scale megawatt power plants in rural areas. SOFCs also allow flexible operation with various fuels, such as hydrocarbons, coal syngas, or impure hydrogen [85]. Herein, we have discussed various perovskite materials for SOFC cathodes and anodes, and suggest effective and stable alternatives for the reliable operation of SOFCs.

**Figure 7:**
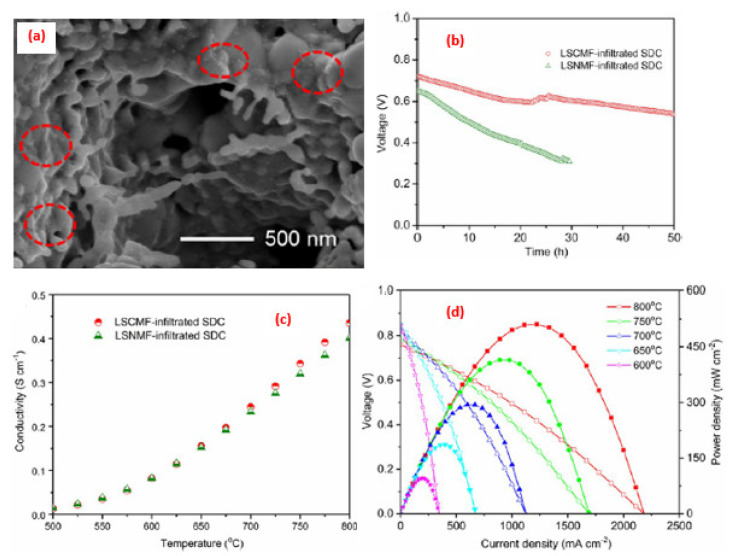
(**a**) EM image of FeCo alloy and LSMF nanoparticles; (**b**) *I*–*V* and *I*–*P* curves of the fuel cells with reduced La_0.6_Sr_0.4_Co_0.2_Mn_0.2_Fe_0.6_O_3−δ_-infiltrated Sm_0.2_Ce_0.8_O_1.9_ 200 ppm of H_2_S-H_2_ at 600–800 °C; (**c**) electrical conductivities of the reduced LSCMF and La_0.6_Sr_0.4_Ni_0.2_Mn_0.2_Fe_0.6_O_3−δ_-infiltrated SDC anodes in 10 vol% H_2_–Ar at 500–800 °C; and (**d**) stability tests of the single cells with FeCo/RP-La_1.2_Sr_0.8_Mn_0.4_Fe_0.6_O_4−δ_ and FeNi/RP-LSMF infiltrated SDC anodes operating at 200 ppm. Copyright 2020 by the American Chemical Society [86].

A detailed comparison of Ruddlesden-Popper-type perovskites adorned with FeNi and FeCo alloy nanoparticles as sulfur-tolerant anodes for SOFCs was explored by Li et al. [86]. Their findings showed that the Fe_3_Co_2_/RP-La_1.2Sr0.8_Mn_0.4_Fe_0.6_O_4−δ_ anode had an extraordinarily superior efficiency, with peak power densities of 632 mW cm^−2^ in H_2_ and 566 mW cm^−2^ in 200 ppm of H_2_S−H_2_ at 800 °C, and a good operating durability of approximately 50 h when operated at 200 ppm H_2_S-H_2_ at 800 °C (Figure 7), thus promoting the commercialization of SOFC technology.

**Figure 8:**
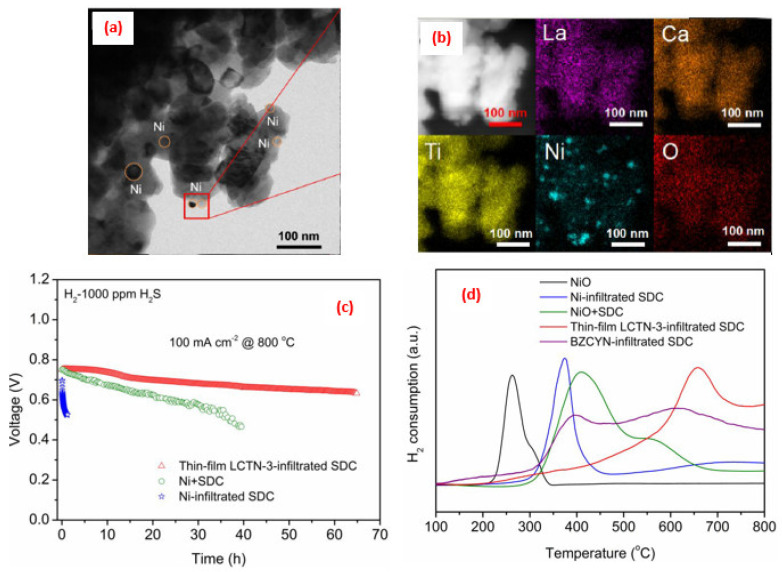
(**a**) Transmission Electron Microscopy image of reduced La_0.33_Ca_0.47_Ti_0.94_Ni_0.06_O_3−δ_ powder; (**b**) Scanning transmission electron microscopy-Energy dispersive X-Ray results of reduced LCTN–3 powder; (**c**) stability test of the fuel cells with Ni-infiltrated SDC, Ni + SDC, and reduced thin-filmLCTN–3-infiltrated SDC anodes operating on H_2_–1000 ppm H_2_S; and (**d**) H_2_-TPR profiles of the Ni-infiltrated SDC, NiO + SDC, thin-film LCTN–3-infiltrated SDC, and Ba(Zr_0.4_Ce_0.4_Y_0.2_)_0.8_Ni_0.2_O_3−δ_-infiltrated SDC anodes and NiO. Copyright 2018 by the American Chemical Society [92].

For the first time Kim et al. [87] have discovered a cobalt-free (Ba_1−*x*_Nd*_x_*FeO_3-δ_) perovskite electrode catalyst can act as a next generation SOFC application, the achieved power output value of 1.2 W cm^−2^ @ 800 °C. The LST surface/interface highways have shown a new wave of high performance SOFCs at low temperatures, such as 908.2 mW cm^−2^ at 550 °C. The present work is a novel approach to develop and create innovative materials and devices for LT-SOFCs, helping to exploit the apparent benefits of the resulting low cost for commercialization. Niobium-doped SrTiO_3_ was tested as a stable redox anode material for SOFCs [88]. The electrode materials exhibited favorable redox stability characteristics, and hence a possibility for the use of Nb-doped strontium titanates as part of the SOFC anode. Single-phase SmBa_0.5_Sr_0.5_Co_2_O_5+δ_ unveiled metallic behavior, with electrical conductivities of 1280 Scm^−1^ at 50 °C and 280 Scm^−1^ at 900 °C. It also revealed half-cell area specific resistances of 0.10 and 0.013 Ω cm^2^ at 600 and 700 °C, respectively; and cell power densities of 1.06 W cm^−2^ at 750 °C, 0.75 W cm^−2^ at 700 °C, and 0.46 W cm^−2^ at 650 °C. The abovementioned results indicate that the SBSCO composite could be used for IT-SOFC applications [89]. Double-perovskites Sr_2_MMoO_6_((M) Co, Ni) have been studied as an anode material for a solid-oxide fuel cell. As a result, Sr_2_CoMoO_6_ had a high power density of 735 mW/cm^2^ in H_2_ and 527 mW cm^−2^ in wet CH_4_ at 800 °C, whereas Sr_2_NiMoO_6_ had an remarkable energy output only in dry CH_4_ [90]. A study by Adijanto et al. showed that Pd@CeO_2_ nanocomposites demonstrated outstanding electrochemical efficiency when using either H_2_ or CH_4_fuels at 973 K. The findings indicated that high stability is anticipated for SOFC function using this precursor at 973 K [91]. Song et al. adopted a new approach to design a composite La_0.35_Ca_0.50_TiO_3_−δ__/SDC anode using a combination of infiltration and in situ exsolution. The composite was found to exhibit higher PPDs and excellent electrochemical activity in H_2_ and H_2_−1000 ppm H_2_S fuels (Figure 8).

It also has good CO_2_ tolerance, indicating the potential of using the material in hydrocarbon-fueled SOFCs [92]. Hibino et al. analyzed the efficiency of a single-chamber solid-oxide fuel cell using a ceria-based solid electrolyte at temperatures below 773 K. The results suggested that the SOFC prepared in their analysis had numerous advantages over PEFCs. First, the anode was not exposed to carbon monoxide poisoning, while this is a serious concern for PEFCs. Second, in their SOFC, there were no noble gases like Pt, so production costs were low. Third, the hydrocarbon reformer would work at a higher temperature than their SOFC, while the PEFC itself can function at ambient temperatures. Finally, there was a more compact cell stack provided by the single-chamber cell configuration. These potential benefits drastically improved the role of SOFCs in the near future as the preferred technique for developing electric power for automobiles [93]. Hui and his coauthor [94] tested yttrium-doped SrTiO_3_ as an anode material for solid-oxide fuel cells in terms of specific conductance, phase stability, oxidation/reduction activity, chemical compatibility with yttria-stabilized zirconia and La_0.8_Sr_0.2_Ga_0.8_Mg_0.2_O_2.8_, coefficient of thermal expansion, and fuel cell efficiency. The researchers noted that when SYT was used as the support system in SOFC membranes, the small grain size could deliver better mechanical characteristics. Double perovskite oxides composed of Sr_2_Mg_1−*x*_Co_*x*_MoO_6−δ_ have been synthesized by Xie et al. using a basic citrate process. The incorporation of Co tuned the parameters of Sr_2_Mg_1−*x*_Co*_x_*MoO_6−δ_ such as improving the electrical and ionic conductivity of Sr_2_MgMoO_6−δ_ at the Mg site, decreasing the forbidden gap of Sr_2_MgMoO_6−δ_, and enhancing the mechanism of SMMO anode charge transfer, thereby reducing the electrode polarization. The authors proposed that the efficiency of the cell could be further enhanced by improving the electrode microstructure and the electrolyte thickness. Moreover, SMCMO is a promising anode material for SOFCs [95]. Jun et al. suggested that the development of ordered perovskite cathodes is necessary for the creation of high-efficiency, highly reliable and stable SOFC activity [94,96]. The SOFCs’ performances and their related parameters are summarized in Table 3.

Materials are often among the first factors in developing low-temperature solid-oxide fuel cells. Liu et al. studied the multi-functionality of a layered perovskite oxide La_2__−*x*_Ce*_x_*CuO_4_ and the authors showed that it was being used as a cathode and anode, and in the electrolyte, enabling it to be used in a single-layer SOFC to simplify the SOFC structure and manufacturing process [97]. The crystalline nature, heat transfer rate, ionic properties, and cathodic polarization of Sm_0.5_Sr_0.5_Fe*_x_*Co_1−*x*_O_3−δ_ (SSFC) and Sm_0.5_Sr_0.5_Mn*_x_*Co_1−*x*_O_3−δ_ (SSMC) were studied by Lv et al. [96,98]. Experimental data showed that the Sm_0.5_Sr_0.5_Fe_0.8_Co_0.2_O_3−δ_ electrode displayed significant catalytic performance for oxygen reduction at temperatures between 700 and 800 °C. It will therefore be a promising candidate for the IT-SOFC cathode material. Materials used as a solid-oxide fuel cell (SOFC) anode must comply with a range of specific requirements. They must have high electron transport and appropriate catalytic activity against the electrode surface reaction to minimize the loss of polarization. Interestingly, Marina et al. studied the thermal, electrical, and electrocatalytic properties of LaSr_1−*x*_TiO_3_ perovskite, and the findings of the analysis revealed that the sample sintered in air had electrical conductivity of 1−16 S/cm, whereas the sample sintered in hydrogen at 1650 °C showed 80−360 S/cm under experimental conditions usual for SOFC anode operation. Additionally, cell studies have shown that doped titanates have had the prospective ability to be used as SOFC anodes [97,99]. Niu and his coauthors conducted a comparative study of the Bi_0.5_Sr_0.5_FeO_3−δ_ (BSF) and La_0.5_Sr_0.5_FeO_3−δ_ (LSF) oxygen reduction reactions as cathodes for intermediary temperature solid-oxide fuel cells. In their study, the authors observed that BSF exhibits strong electrochemical properties that can be further streamlined by improving its ionic conductivity. Self-assembly recently became an evolving technique for preparing composite cathodes with strong electrochemical oxygen-reduction operation and congenital chemical stability for solid-oxide fuel-cell intermediate temperatures [100]. Qi et al. prepared a BaZr_0.6_Co_0.4_O_3−δ_ perovskite nanocomposite, and it has been tested as a cathode for the moderate-temperature solid-oxide fuel cell. Hexagonal 12H-BaCo_0.96_Zr_0.6_Co_0.04_O_2.6+δ_ and cubic BaZr_0.82_Co_0.18_O_3−δ_ nanocrystals aggregated with pomegranate-like particles that substantially improved the three phase-boundary sites for oxygen reduction. Moreover, a single-cell anode assisted by a BaCo_0.6_Zr_0.4_O_3__−δ_ nanocomposite cathode achieved a high power density of 1530 mW cm^−2^ at 750 °C and 414 mW cm^−2^ at 550 °C. It is known that various layered perovskite-related oxides show significant electronic, magnetic, and electrochemical properties [101]. For the first time, Sengodan et al. showed that the layered materials would be used as high-performance fuel electrodes. The authors fabricated the PrBaMn_2_O_5+__δ_ node by in situ annealing of Pr_0.5_Ba_0.5_MnO_3−δ_. At 800 °C, PBMO showed a high electrical conductivity of 8.16 S cm^−1^ in 5% H_2_ and a peak power density of 1.7 and 1.3 W cm^−2^ at 850 °C using humidified hydrogen and propane fuels, respectively [102]. Shaheen et al. synthesized a novel and effective Sr_0.4_La_0.6_(Fe_0.75_Ti_0.25_)_0.6_Ni_0.4_O_3__−δ_ nanocomposite and tested SOFCs based on multi fuels. The results indicated that the synthesized nanocomposite was well suited for low-temperature multi fuel SOFCs that are cost-effective [103]. Godickemeier et al. studied the impact of high oxygen diffusivity on the kinetics of oxygen reduction. The authors noted that La_0.8_Sr_0.2_MnO_3_ acted as a model material for cathodes, with high oxygen reduction activity and low oxygen ionic conductivity. Composites composed of silver and bismuth vanadates demonstrated exceptional catalytic properties at 500−550 °C for oxygen reduction, and significantly minimized the resistance of low-temperature SOFCs to cathode–electrolyte interfacial polarization, down to around 0.53 Ω cm^2^ at 500 °C and 0.21 Ω cm^2^ at 550 °C. The recorded power densities of 231, 332, and 443 mW cm^−2^ at 500, 525, and 550 °C, respectively, allowed the activity of SOFCs at temperatures of approximately 500 °C. A substantial reduction in operating temperature can greatly reduce not only material costs, but also manufacturing costs. It also ensures improved device stability, longer functioning life, and increased capacity for smart-phone applications [104]. Low-temperature SOFCs have enormous potential to be affordable for many uses, including residential and automotive applications [105]. In their analysis, Niu et al. found that Bi doping of SrFeO_3−δ_ induced the creation of a high symmetry structure and excellent electrochemical efficiency for Bi_0.5_Sr_0.5_FeO_3−δ_, which can compete effectively with the Co-based cathode, with further advantages of lower thermal expansion and cost [104,106]. LT-SOFCs have recently received huge worldwide exposure with a new research pattern based on single-layer fuel cells. In this context, Jhuang et al. used the triple-conductive perovskite BaCo_0.4_Fe_0.4_Zr_0.1_Y_0.1_O_3−δ_ as an intermediate layer for SOFCs, which indicated a current density of 994 mA cm^−2^ at 0.6V and a power density of 610 mW cm^−2^, with an OCV of 1.01 V at a cell operating temperature of 550 °C, thereby confirming the feasibility of using BCFZY in SLFCs [107]. High-temperature, zirconia electrolyte-based, yttria-stabilized SOFCs provide many benefits over conventional energy systems. These include high efficiency of conversion, durability, modularity, adaptability to fuel, and nearly infinite sustainability due to very low emissions of NO*_x_*. In addition, these cells create high-quality exhaust heat that can be used to further improve total plant productivity for process heat or a bottoming electrical power cycle. Once completely commercialized, SOFC systems are intended to support a wide variety of electricity and heat applications, such as huge power generation by electrical and gas utilities, and industrial cogeneration [108]. An extremely effective, pollution-free energy-production technology is provided by high-temperature solid-oxide fuel cells. Their efficiency has been proven through successful activity in a range of atmospheric-pressure-generating systems up to 100 kW in scale.

## 6. Perovskite-Based Nanocomposites in Battery Studies

**Figure 9:**
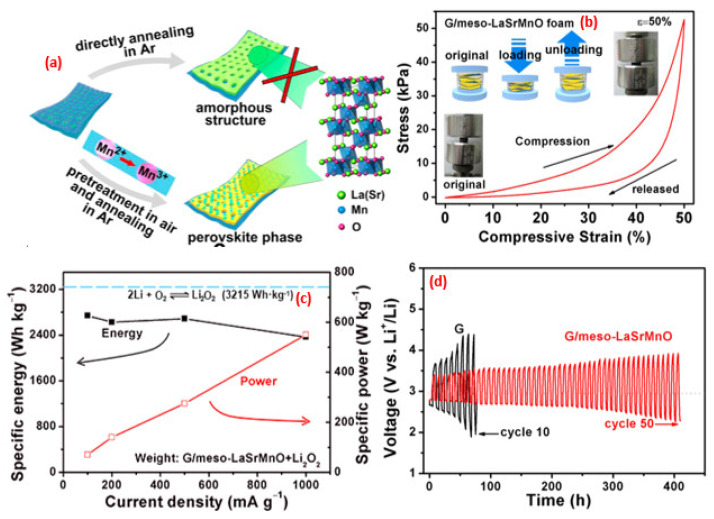
(**a**) Schematic illustration of the formation process of perovskite structure under different reaction conditions. (**b**)G/meso-LaSrMnO foam at a specific strain of 50%. The inset shows the structural model of the meso-LaSrMnO monolith and G/meso-LaSrMnO foam. (**c**) The specific energy and power of the Li-O_2_ cell with a G/meso-LaSrMnO electrode. (**d**) Voltage versus time graph of G and G/meso-LaSrMnO electrode at a discharge limit of500 mAh·g^−1^. Copyright 2016 by the American Chemical Society [110].

Kostoet al. showed CsPbBr_3_ micro cubes with improved electrochemical performance; these can be the best anodes among the nano- and micro particulate lead halide perovskite anodes used for Li-air batteries, to date. Lithium-rich, anti-perovskite superionic conductors are an extremely interesting class of materials with potential applications as solid electrolytes in Li-ion batteries [109]. Perovskite is used in Li-O_2_ cells as a low-cost catalyst. The two main factors that affect the production of high-performance electrodes are: (i) the limited porosity, which prohibits the transport of molecules; and (ii) the low electronic conductivity (Figure 9). Mesoporous LaSrMnO in graphene foams was synthesized by Yang et al. via a facile soft chemistry method, and was assembled a 3D hierarchical architecture. The G/meso-LaSrMnO foam exhibited an improved oxygen reduction reaction and oxygen evolution reaction catalytic stability, good specific capacity, high rate capability, and cyclic stability. Therefore, the high catalytic mesoporous perovskites, in combination with the conductive graphene networks, constitute a beneficial strategy for the development of effectual electrodes in numerous energy-storage systems [110].

**Figure 10:**
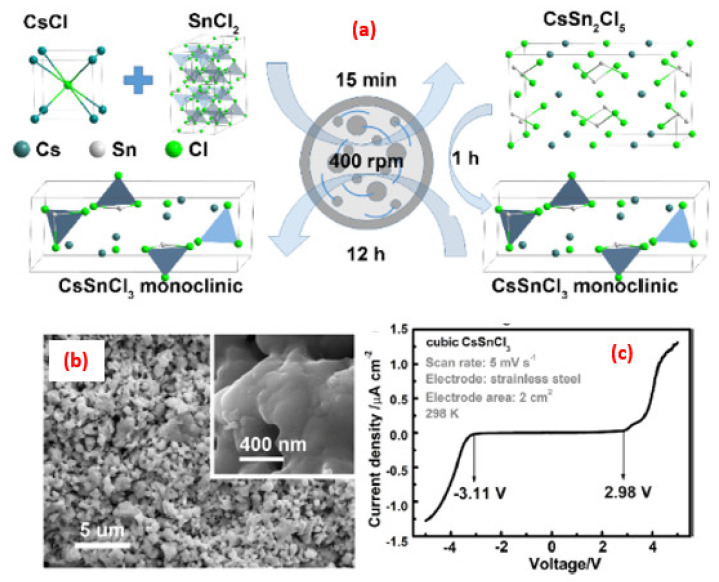
(**a**) Schematic figure shows the fabrication process of the monoclinic CsSnCl_3_ material; (**b**) SEM image of the as-prepared monoclinic CsSnCl_3_ material; and (**c**) Linear sweep voltammetry scan (5 mV s^−1^) of the cubic MM−1 h-HT electrolyte at 298 K. Copyright 2020 by the American Chemical Society [111].

A room-temperature-stable inorganic halide perovskite of CsSnCl_3_ has been studied for its potential as a solid electrolyte in CIB applications. The electrolyte showed a low electronic conductivity of 2.17 × 10^−10^ S cm^−1^ and a large electrochemical window of about 6.1 V at 298 K (Figure 10).

The XPS and CV results confirmed that the prepared cubic CsSnCl_3_ electrolyte was a chloride ion conductor [111]. To maximize the Li^+^ conductivity in the lithium-rich anti-perovskites (LRAPs), Ong et al. adopted a rational composition strategy guided by a combination of first-principle calculations and percolation theory. AIMD simulations showed a higher conductivity for Li_3_OCl_0.75_Br_0.25_ as compared to Li_3_OCl_0.5_Br_0.5_, and this was the highest composition of conductivity in the anti-perovskite chemistry identified experimentally so far [112]. The sol-gel method is a traditional method for synthesizing perovskites; however, small surface areas of the perovskites synthesized by this method lead to low catalytic site utilization, which further reduces the performance of Li-O_2_ batteries and limits its application [113].Fascinatingly, a hierarchical mesoporous/macro porous perovskite La_0.5_Sr_0.5_CoO_3−*x*_ nano tube was designed to promote its use in Li-O_2_ batteries. The excellent performance of Li-O_2_ batteries was attributed to the synergistic effect of the high catalytic activity and the stable structure of the perovskite-type HPN/LSC. Moreover, this hierarchical meso porous/macro porous nano structured perovskite-type catalyst can be used for the future development of high-performance Li-O_2_ batteries. Yan et al. designed MnO_2_/La_0.7_Sr_0.3_MnO_3_ hierarchical core-shell composite materials by the selective dissolution method. The prepared MnO_2_/La_0.7_Sr_0.3_MnO_3_ materials showed good catalytic activity toward ORR/OER, and were thus used as bi-functional oxygen electrocatalysts for metal–air batteries [114]. Bu et al. demonstrated the effects of the cation-ordered perovskite oxide PrBa_0.5_Sr_0.5_Co_2−*x*_Fe*_x_*O_5+δ_ as a highly active and stable catalyst for OER and ORR in alkaline media. The enhancement of the catalytic efficiency of PBSF-NF was attributed to the mesoporous nanofiber structure, high electrical conductivity, swift oxygen kinetics, and structural permanence. These promising findings have led to a significant improvement in the performance of Zn air batteries and the reliability of their charge. Hence, PBSCF-NF mesoporous nanofiber is a hopeful catalyst for rechargeable metal–air batteries [115]. Interestingly, Chen et al. introduced a highly active and durable bi-functional composite catalyst, intertwined core–corona structured bifunctional catalyst to utilize in rechargeable metal–air battery applications. The prepared composite had a strong inter particle interaction that led to fast charge transfer and diffusion of reactants during the electrochemical oxygen reactions. Additionally, this study illustrated the utilization of highly engineered composite morphology structures to improve catalytic operation and longevity for the commercialization of high-performance rechargeable metal–air batteries [116]. Yue et al. synthesized perovskite-type CeMnO_3_ nano fibers via the electro spinning method. They observed that the prepared nanofibers had a rough surface and mesoporous structure. The CeMnO_3_ anode had a discharge capacity of 2159 mA hg^−1^ with a coulombic efficiency of 93.79%. As a high reversible capacity of 395 mA hg^−1^ at 200 mA g^−1^ for CeMnO_3_ was obtained at a high discharge rate of 1000 mAg^−1^ after 60 cycles, this nanofiber could be a promising anode material for lithium-ion batteries [117]. Tong et al. synthesized novel perovskite-type silver molybdenum oxy-fluorides via a mechano-chemical reaction. As cathodes, the silver molybdenum oxy fluoride perovskites showed good electronic conductivity and electrochemical activity, even without the inclusion of carbon black for lithium batteries [118]. The small particles with a wide surface area are essential to improve catalytic efficiency, but the disadvantage of better reactivity is that the compounds are less stable. If the size of the particles is too small, thermal or chemical treatments annihilate the structure, such as the growth of carbon nanotubes. With the developed synthesis methods, it is possible to design the particle size and surface structure of the metal oxide particles to have both a maximum surface area and a stable structure. This development can be applied to increase the efficiency of Zn/air batteries [119]. LaCoO_3__−δ_ (LC) base metal-organic framework (MOF) electrode is being investigated for their application in SOFCs, moreover, the multi-active Co-MOF/LC is an extremely promising electrode catalyst in rechargeable Zn-air batteries [120].Perovskites are of vital importance as a substitute for precious metals and oxides used in bi-functional air electrodes involving the oxygen evolution reaction and oxygen reduction reaction. As observed by Hardin et al., LaNiO_3_ nanocrystalline aggregates display high OER activity and good OER/ORR bifunctional character, which is vital for the development of inexpensive metal–air batteries, fuel cells, and electrolyzers [121]. Perovskite materials of the ABO_3_ type have greater catalytic activity relative to binary metal oxides because of crystallographic defects and oxygen vacancies due to the multivalence of the A and B sites [122]. For the first time, Kim et al. developed ortho rhombically distorted perovskite SeZnO_3_ with lithium oxygen battery as the electrocatalysts. Due to the valence effects of Se^4+^ and Zn^2+^, the structure of the perovskite was crystallographically tilted and distorted from defects and oxygen vacancy, which gave structural advantages as an electrocatalyst for the O_2_ electrode [123]. A comparative study conducted by Lu et al. showed that NiTiO_3_ powder synthesized using a rapid microwave-assisted solvo-thermal process crystallized in a pure rhombohedral phase, whereas the impurity phase of TiO_2_ was observed for the NiTiO_3_ powder prepared by the conventional solvothermal process. The NiTiO_3_ powder synthesized using the microwave-assisted method resulted in a high specific discharge capacity of 750 mAh/g at 0.1 C rate, 90% columbic efficiencies, and a charge-transfer resistance of 16 Ω. The reduction in the charge-transfer resistance improved the movement of Li_+_ ions due to the rapid reaction of the microwaves. As a result, the battery impedance decreased and the reversibility of the battery increased. Eventually, the authors concluded that the microwave-assisted solvothermal method could also be a facile approach for the preparation of the NiTiO_3_ anode material for lithium-ion batteries. Recently, researchers have found that doping can significantly improve the catalytic performance of perovskite oxides [123]. The research findings of Lv and his coworkers showed that the perovskite-type LaMn_0.8_Cu_0.2_O_3_ and LaMn_0.8_Co_0.2_O_3_ had good bi-functional catalytic activities due to the presence of Mn^4+^/Mn^3+^ and oxygen vacancies. The study exemplified that B-site doping is an effective solution to improve the ORR and OER catalytic activity of perovskite in Li-O_2_ batteries [124].Many researchers have shown that the catalytic activity of the ABO_3_ perovskite toward the oxygen reduction reaction and oxygen evolution reaction is enhanced while tuning the A site’s deficient and excessive stoichiometry [125]. In a study, Miao and his coworkers chose the simplest Mn-based perovskite (LaMnO_3_) to explain the deficient effects and enhancing mechanisms on both ORR and OER. The results exhibit that A-site-deficient stoichiometry is more favorable to the catalytic activity and stability of LaMnO_3_ toward both ORR and OER than A-site-excessive stoichiometry. Consequently, A-site-deficient, Mn-based perovskite could be used for aqueous and solid-state flexible zinc–air battery applications. Electric vehicles that utilize a lithium-ion battery pack for propulsion have gained a great deal of attention [125]. Interestingly, Xu et al. studied the use of perovskite solar-cell packs with a LiFePO_4_ cathode and Li_4_Ti_5_O_12_ anode. The results of the study showed that the device had high photoelectric conversion, 7.80% storage efficiency, and remarkable cycling stability, and hence holds promise for various possible applications [126]. Zhang et al. studied the perovskite-structured titanate La_0.5_Li_0.5_TiO_3_ to include this in the family of anode materials. The anode showed a specific capacity of 225 mA hg^−1^ and sustained 3000 cycles while involving a reversible phase transition with average potential of around 1.0 V vs. Li^+^/Li. Moreover, the rate performance exceeded the nano structured S_12_ without reducing the particle size from micro- to nanoscale [127]. The perovskite oxide La_0.6_Sr_0.4_Co_1−*x*_Ni*_x_*O_3_ (*x* = 0, 0.05, 0.1) was synthesized using a dry gel combustion method [128]. It was observed that when Ni content increased, surface oxygen species also increased, which corresponds to the formation of more oxygen vacancy. Additionally, among the three catalysts studied, La_0.6_Sr_0.4_Co_0.9_Ni_0.1_O_3_ showed the best catalytic activity. The statistics of the specific capacity parameters with various nanocomposite electrodes are summarized in Table 4.

The oxygen reduction reaction is an important process in electrochemical conversion and storage devices. A highly active nonprecious metal containing oxide catalysts could be developed for oxygen reduction by tuning the surface electronic features such as transition metal e_g_ filling and covalency [129].For lithium–oxygen battery applications, the biggest challenge is the fabrication of efficient bifunctional catalysts for the oxygen reduction reaction and oxygen evolution reaction in cathodes. The electrospinning method was used by Wang et al. to synthesize Sr_0.9_Y_0.1_CoO_3−δ_ perovskite nanorods. The study results proved that the perovskite Sr_0.9_Y_0.1_CoO_3−δ_ is an effective bifunctional electrocatalyst for lithium–oxygen batteries, and the impregnation of CoO nanoparticles can be an effective approach to improve the cathode performance as well [130].

Perovskite batteries are highly preferred by researchers because of their distinctive advantages. Nanoperovskite, or K(Mn_0.95_Ni_0.05_)F_3_, was studied by Wang et al., was synthesized using an Ethylenediamine tetraacetic acid-assisted homogeneous precipitation method. In addition, to enhance the electron conductivity of the electrode materials, the authors deposited the solid-state material on multi-walled carbon nanotubes to form K(Mn_0.95_Ni_0.05_)F_3_/MWCNT nanocomposites that show charge–discharge capacities of 106.8 and 98.5 mA h g^−1^ after the 60th cycle over a voltage range of 4.2−1.2 V vs. K/K^+^ at a current density of 35 mA g^−1^, respectively [131]. Xu et al. prepared a La_0.75_Sr_0.25_MnO_3_ -LSM catalyst that elevates the round-trip efficiency by suppressing the ORR and OER over potentials. The hollow channel structure and good catalytic activity provided high cyclic stability, rate capability, and specific capacity to the Li-O_2_ cells [132]. Xu et al. prepared the perovskite oxide Ba_0.9_Co_0.7_Fe_0.2_Nb_0.1_O_3−δ_and applied it in lithium–oxygen batteries as the oxygen electrode catalyst. Using 80% Ketjen black and 20% BCFN9721 as an oxygen electrode showed the best performance, and the cell could run for 24 cycles devoid of any capacity delay. Based on the observed results, the authors concluded that the Ba_0.9_Co_0.7_Fe_0.2_Nb_0.1_O_3−δ_perovskite oxide could be used as a promising bi-functional oxygen electrode catalyst for rechargeable Li–oxygen batteries [133]. La_0.7_Sr_0.3_MnO_3_ and (La_1−*x*_Sr*_x_*)_0.98_MnO_3_ (*x* = 0.2–0.5) perovskites with Sr as the dopant were studied by Xue et al. [134]. The impregnation of Sr with La and introducing the A-site deficiencies can successfully tailor the Mn valence and O species in LSM perovskites. Among the samples studied, the (La0.7Sr0.3)_0.98_MnO_3_ composited with 50% carbon showed good ORR catalytic activity, which was due to the excellent oxygen adsorption capacity. Moreover, the aluminum–air battery, which uses 50% LSM30, exhibited the maximum power density, and can be used as the oxygen reduction reaction catalysts in metal–air batteries.

## 7. Perovskite-Based Solar-Cell Studies

The rapid development of fourth-generation perovskite-based solar cells (PSCs), which meet energy requirements and could be harvested with unique natures like abundance, eco-compatibility, and low-cost fabrication, explore the conversion of solar energy in to electrical energy [135]. In general, this new type of PSC technology has received increasing attention due to the light-harvesting active layer composed between the electron transport layer (ETL) and hole-transport layer (HTL) [136]. Despite this, the study showed the major development of PSCs, which are addressing the issues (degradation mechanism) of device stability while optimizing the solar-cell efficiency [137]. Thereby, using novel and lower-cost mesoporous morphology of methyl ammonium lead halide-based TiO_2_/CH_2_NH_3_PbI_3_/HTM/Au electrode, when being employed in PSCs, they achieved PCSs of 8.38% [138]. The PCEs of various perovskite-based anode materials are summarized in Table 5.

**Figure 11:**
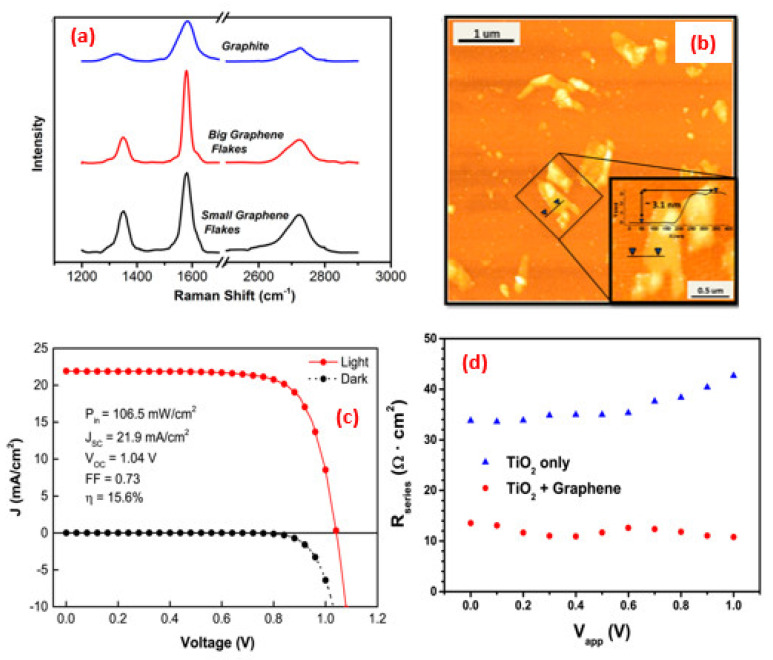
(**a**) Raman spectra of starting graphite flakes and exfoliated graphene deposited on the SiO_2_ substrate with increasing sonication time. (**b**) AFM image of exfoliated graphene scanned at 5 μm × 5 μm and enlargement of the graphene flakes. (**c**) The best-performing (*η* = 15.6%) solar cell based on graphene–TiO_2_ nanocomposites under simulated AM1.5, 106.5 mWcm^−2^ solar irradiation (solid line), and in the dark (dotted line), which were processed at temperatures not exceeding 150 °C. (**d**) Series resistance R series obtained from impedance spectroscopy analysis of samples with two different concentrations of graphene mixed with TiO_2_ in the electron collection layer. Copyright 2014 by the American Chemical Society [139].

The solution-based fast-printing deposition of an emerging high-quality nanoflake-structured, graphene-supported TiO_2_ (graphene/TiO_2_) nanocomposite has been intensively explored as a low-cost solar cell due to is suitable superior charge-collection with a remarkable high-efficiency PCE of 15.6% (Figure 11) [139].

**Figure 12:**
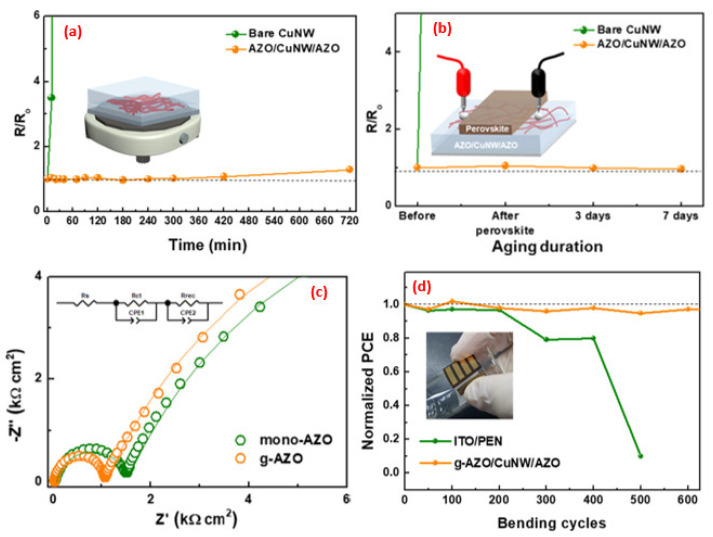
Sheet-resistance variation (**a**) at ambient conditions of 120 °C for the bare CuNW film and AZO-based composite electrode as a function of the annealing time; (**b**) annealing after the spin-coating of the perovskite precursor solution; (**c**) Nyquist plots of flexible PSCs with different electrodes obtained using IS; and (**d**) normalized PCEs of flexible PSCs with indium tin oxide/polyethylene naphthalate and g-AZO-based composite electrodes. Copyright 2020 by the American Chemical Society [140].

The flexible and cost-effective Al-doped gradient ZnO layered copper nanowire (g-AZO/CuNW/AZO) composite was fabricated using the atomic layer deposition method, and the resulting protected AZO/CuNW flexible PSC exhibited a PCE of 14.18% and maintained the reasonable initial PCE after 600 bending cycles (Figure 12) [140].

Solution-based silver nanowire (AgNWs) PSCs offer an ultrathin transparent active layer, which provides significant potential PSC applications with a reported PCE of 11.00% efficiency [141]. The bi-functional nature of gold nanoparticle-modified polymer nanocomposite (poly(3-hexylthiophene-2,5-diyl)) (P3HT:AuNPs) has become a promising energy-storage device for efficiently harvesting PSC, with are ported PCE of 10.71% efficiency [142]. Table 5 shows some PCE parameters of perovskite-based composites [143,144,145,146,147,148,149,150]. A highly efficient hole-transport material (HTM)-based perovskite (2,2′,7,7′-tetrakis-(N,N’-di−4-methoxyphenylamino)-9,9′-spirobifluorene (spiro-OMeTAD) printable porous counter electrode has also been developed, with a most notable PCE of 3.34% [151]. One-dimensional TiO_2_ nano column arrays have recently gained attention and are considered as a more efficient future designed solar cell, with an exhibited PSC of 7% [152]. Hvojnik et al. fabricated a TiO_2_-blocking supported carbon-black PSC device, which showed a better PCE of 7.8% [153]. TiO_2_/Gr nano composites are a new class of solar-cell materials, and can also be used as an sustainable electron transport layer in this connection to increase carrier transport with enhanced PCE of 17.94% [154]. The smooth morphological structure of the mixed (tin/lead iodide) perovskite electrode is the most promising double hole-transport layer-based photovoltaic technology, and efficiently displayed their PCE value [155]. The uniform meso porous hybrid structure of a TiO_2_/Graphene/CNT composite has been extensively studied as the photo anode, which has superior charge-transport properties and enhanced noticeable PCEs up to 13.97% [156]. A highly transparent, conductive with electron-transport-layered, MoS_2_-based, triethylenetetramine-doped graphene (TETA-GR) composite has been fabricated by the spin-coating method, and could represent an efficient photovoltaic enhancement [157]. Most importantly, a graphitic carbon-modified SnO_2_ (SnO_2_/g-C_3_N_4_) nanocomposite could regulate the charge-extraction behavior and energy-band alignment to promote the PCE (22.13%) and long-term device stability [158]. The mesoporous structures of rGO/TiO_2_ nanocomposites have attracted research efforts due to their charge-collection efficiency, open circuit voltage, and charge-current density [159]. TiO2/graphene nanocomposites can be used as a mesoscopic PSCs, which is mainly due to significantly improving their PCE with fill factor [160].

## 8. Electrochemical Measurements and Cyclic Stability

**Figure 13:**
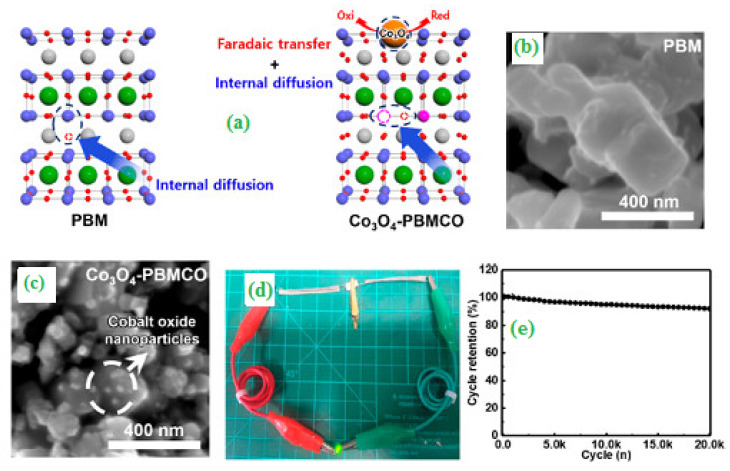
(**a**) Schematic of energy storage mechanism for PBM and Co_3_O_4_−PBMCO; (**b**) SEM image of PBM; (**c**) HR-TEM images of Co_3_O_4_-PBMCO; (**d**) digital-camera image of a green LED powered by two wire-type hybrid devices connected in series; and (**e**) cycling performance of the hybrid device for 20,000 cycles. Copyright 2020 by the American Chemical Society [164].

Recently, perovskite-based electrode materials were used as a novel matrix that holds great potential for the development of high energy density, high power density, and long cyclic durability [161].Although a challenge has been made to improve the electrochemical capacitance properties of Gd_2_NiMoO_6_ perovskite via the facile wet chemical route, the relevant perovskite electrode materials demonstrated high specific capacitance (400.46 F g^−1^) with an applied current density value of 1A g^−1^ [162]. Herein, Li_3*x*−*y*_La_1−*x*_Ti*_x_*O_3_ type perovskite-based solid electrolytes have been used as the base materials for Li-ion conductivity (4.53 10^−4^ S cm^−1^), with discharge capacity value of 107.6 mA hg^−1^ [163]. Kang et al. [164] developed the 3D-layered structured PrBaMn_1.7_Co_0.3_O_5+δ_ (Co_3_O_4_-PBMCO) composite as a promising reference active material. It had vacancy-accelerated properties with outstanding specific capacity (1571 F g^−1^), and good cyclic stability (Figure 13).

Kitchamsetti et al., [165] used meso porous interlocked nickel titanate (NTO) rod-based perovskite for fascinating materials in a supercapacitor application, and showed a unique rod morphology and significantly better capacitance properties (542.26 F g^−1^) with better cyclic stability (2100 cycles). Using cyclic voltammetry and the charge–discharge method, Pious et al. [166] prepared an 0D (CH_3_NH_3_)_3_Bi_2_I_9_ lead-free thin-film electrode as a potential perovskite electrode material for electrochemical double-layer capacitors (EDLC). Specifically, the electrochemical behavior of the MAPbI_3_ electrode demonstrated an areal capacitance value of 5.5 mF cm^−2^ and charge–discharge cyclic stability of up to 10,000 cycles.

## 9. Conclusions

In this review, we successfully highlighted the recent developments in a new class of high-performance-based perovskite electrode catalysts for electrochemical studies. These can be used as a new class of potential candidates for large-scale production of energy devices (supercapacitors, batteries, fuel cells, solid-oxide fuel cells, and solar cells). The different synthetic strategies, unique structural morphologies, and advanced analytical techniques have been pointed out as part of a benchmark analysis for improving future energy catalytic activity. Hence, the layered perovskite structures exhibited outstanding electrocatalytic activity, which could be discussed as a new idea for improving the power output with long-term electrode stability. The recent desirable development of advanced electrode catalysts, which are very promising for high-efficiency modules, can ensure fast electrontransfer and maximum power efficiency with environmental safety.

## Figures and Tables

**Table 1:** Comparison of different perovskite-based nanocomposites for supercapacitor applications.

Synthesis Strategies	Materials	Typical Morphology	Specific Capacitance (F g^−1^)	Power Density (W kg^−1^)	Energy Density (Wh kg^−1^)	Cycles	Ref.
Coprecipitation	La_1−*x*_Sr*_x_*BO_3−δ_	-	492	31	10,000	-	[41]
Sol-gel	SrMnO_3_	Nanofiber	446.8	37.3	400	5000	[53]
Sol-gel	NiTiO_4_	Rod	542.26	8.06	4320	2100	[51]
Hydrothermal	BFO-RGO	Nanowire	928.43	18.62	950	1000	[63]
Solid-state	CuO/LSC73	Porous	545	4.92	-	3500	[39]

**Table 2:** Fuel-cell performance characteristics of different perovskite-based nanocomposites.

Synthesis Strategies	Anode Materials	Typical Morphology	Open Circuit Potential (*V*)	*P_max_* (mW cm^−2^)	Stability	Ref.
Solution-casting	La_0.9_Sr_0.1_CrO_3-δ_	Nanoparticles	0.5	62	180 °C	[65]
One-pot Pechini method	Ba-Ce-Fe-Co-O	Grains	1.036	335	700 °C	[67]
Sol-gel	La_0.25_Sr_0.75_TiO_3_	Core-shell	1.11	908.2	550 °C	[75]
Pechini process	(PrBa)_0.95_Fe_1.8−*x*_Cu*_x_*Nb_0.2_O_5+δ_	Porous	1.09	431	800 °C	[76]
Solid phase blending method	CuFeO_2_-LZSDC	Pore	1.1	672	550 °C	[77]

**Table 3:** SOFC performance of various perovskite-based anode materials.

Synthesis Strategies	Anode Materials	Typical Morphology	*P_max_* (mW cm^−2^)	Stability	Ref.
In situ method	La_0.6_Sr_0.4_Mn_0.2_Fe_0.6_O_3−__δ_	Nanoparticles	632	800 °C	[86]
In situ exsolution method	La_0.35_Ca_0.5_0TiO_3−δ_(LCT)/Ba_0.5_Sr_0.5_Co_0.8_Fe_0.2_O_3−δ_ (BSCF)	Core-shell	460	800 °C	[92]
Ball milling	SmBa_0.5_Sr_0.5_Co_2_O_5+δ_	-	131	800 °C	[89]
Tape-casting method	Pd@CeO_2_	Core-shell	30	800 °C	[91]
Solid-state method	Sr_0.88_Y_0.08_TiO_3−δ_	Grains	58	900 °C	[94]

**Table 4:** Performance comparison of specific capacities with different electrode catalysts.

Synthesis Strategies	Materials	Typical Morphology	Specific Capacity (mAh g^−1^)	Stability (Cycles)	Ref
Hummers	G/Meso-LaSrMnO	Nanosheets	6515	50	[110]
Electrospinning	La_0.5_Sr_0.5_CoO_3−*x*_	Nanotubular	500	50	[113]
Electrospinning	CeMnO_3_	Nanofiber	2159	60	[117]
Mechano-chemical	SMOF	-	153	-	[118]
Simple wet chemistry	SeZnO_3_	Nanosheets	13,200	140	[122]

**Table 5:** Photovoltaic characteristics of different electrode catalysts.

Synthesis Strategies	Architectures	Typical Morphology	Power Conversion Efficiency (PCE) (%)	*V_OC_* (V)	Fill Factor (%)	Ref.
Spin-coating	TiO_2_/CH_3_NH_3_PbI_3_/HTM/Au	Core-shell	8.38	0.924	48.7	[138]
Spin-coating	Graphene/TiO_2_	Nanoflakes	15.6	1.04	0.73	[139]
Spin-coating	g-AZO/CuNW/AZO	Nanowire	14.18	1.08	70.7	[140]
Spin-coating	AgNWs	Nanowire	11.0	0.96	0.23	[141]
Spin-coating	P3HT:AuNPs	Nanoparticle	10.71	0.75	64.79	[142]
Spray-pyrolysis	TiO_2_/PbI_2_	Mesoporous	15.00	0.993	0.73	[143]
Physical vapor deposition (PVD)	Nanocolumnan 1D TiO_2_	Nanowire	10.52	0.949	0.64	[144]
Spray-pyrolysis	mp-TiO_2_	Mesoporous	16.2	1.06	0.75	[145]
Spray-pyrolysis	Al-BSF-Si wafer	Hollow squares	19.52	0.693	79.4	[146]
-	Cs_0.1_FA*_x_*PbI_2+*x*_	-	16.6	1.07	80	[147]
Spin-coating	MAPbI_3_	Mesoporous	20.28	1.12	77.7	[148]
Flash evaporation	CH_3_NH_3_PbI_3_	-	12.2	1.07	68	[149]
Vacuum-flash-assisted solution processing	FA_0.81_MA_0.15_PbI_2.51_Br_0.45_	Mesoporous	19.6	1.143	0.76	[150]

## Data Availability

Not applicable.
